# Posterior Cortical Atrophy Masked by a Pituitary Macroadenoma: Diagnostic Challenges in the Setting of Dual Visual Pathology

**DOI:** 10.7759/cureus.99908

**Published:** 2025-12-23

**Authors:** Adam S Bloomfield, Amelia M Mori

**Affiliations:** 1 Geriatrics, Metro North Health, Brisbane, AUS

**Keywords:** diagnostic anchoring, early visual impairment, neurodegenerative disesase, non-secreting pituitary macroadenoma, posterior cortical atrophy, progressive dementia

## Abstract

Posterior cortical atrophy (PCA) is a rare neurodegenerative syndrome characterised by progressive visuospatial and visuoperceptual dysfunction. Diagnosis is frequently delayed, particularly when coexisting visual pathology provides an alternative explanation for symptoms. A man in his sixties with a known pituitary macroadenoma and right homonymous hemianopia presented with a four-year history of insidious visual and functional decline. Prior serial perimetric assessments demonstrated progressive, inconsistent field loss not explained by stable chiasmal compression. Neurological assessment revealed prominent posterior cortical deficits, including features of both Balint and Gerstmann syndromes. MRI demonstrated parietal-predominant cortical atrophy, and fluorodeoxyglucose-18 Positron Emission Tomography (18F-FDG-PET) confirmed marked parieto-occipital hypometabolism, establishing a diagnosis of PCA. This case illustrates how established anterior visual pathway disease can delay recognition of concurrent neurodegenerative pathology. For patients with known afferent visual impairment, clinical indicators to prompt consideration of a cortical pathology and a proposed structural assessment framework are discussed.

## Introduction

Posterior cortical atrophy (PCA) is a neurodegenerative syndrome most commonly associated with underlying Alzheimer’s disease (AD) pathology, accounting for an estimated 5-10% of early-onset AD cases [1-3]. Patients typically exhibit early visual and cognitive impairment with relatively preserved episodic memory and insight. Deficits in visuospatial processing, visuoperceptual function and praxis are common, with constructional apraxia, space perception deficits and simultanagnosia amongst the commonest presenting posterior cognitive dysfunctions [1,4,5]. Diagnostic frameworks stress the need to exclude primary afferent visual disorders, which may otherwise account for the presenting symptoms [2]. Recurrent investigation for eye disease commonly precedes diagnosis of PCA [6], and retrograde trans-synaptic degeneration affecting the retina has been demonstrated in case-control studies [7]. To date, concurrent primary anterior visual pathway lesions have not been reported; we present the first case of PCA coexisting with anterior pathway pathology secondary to a pituitary lesion.

Diagnosing PCA in patients with established anterior pathway disease is therefore challenging. Under such circumstances, clinicians risk misattributing signs and symptoms of PCA to known visual pathology, a form of diagnostic anchoring that may substantially delay recognition [8].

## Case presentation

History

A man in his sixties described a four-year history of functional decline characterised by difficulty reading, navigating text, and performing occupational tasks. A subsequent fall prompted medical evaluation, which revealed a large pituitary macroadenoma compressing the chiasm and left optic nerve on CT brain. Subsequent imaging and pituitary evaluation confirmed stability of the lesion, though serial perimetry demonstrated inconsistent and progressive field loss incongruous with a radiologically unchanged sellar mass.

Four months prior to our evaluation, he was admitted to the hospital with marked deterioration: inability to read or visually scan, cessation of driving, job loss, and infrequent non-threatening visual hallucinations of “small children” within his hemianopic fields. Visual field testing revealed a right homonymous hemianopia with superimposed irregular field loss in the intact left hemifield. There were no other cranial neuropathies, Parkinsonism, or upper or lower motor neuron signs. CT brain identified bilateral hippocampal atrophy in addition to the stable macroadenoma. His Montreal Cognitive Assessment score [9] was 10/30, with impairment across multiple domains, including visuospatial/executive function, language and delayed recall; orientation was relatively preserved.

He was referred to our geriatric evaluation and management unit. Detailed neurological assessment demonstrated both Balint syndrome (oculomotor apraxia, optic ataxia and simultanagnosia) and Gerstmann syndrome (acalculia, agraphia, right-left disorientation and finger agnosia), localising dysfunction to the posterior parietal and parieto-occipital cortices. These findings exceeded what would be expected from a macroadenoma alone.

Investigations

Initial investigations excluded reversible causes of cognitive decline. Routine biochemistry, pituitary screen, thyroid function, vitamin B12, folate, and serology for syphilis and HIV were unremarkable. Autoimmune encephalitis antibody panel and paraneoplastic screen were negative. Cerebrospinal fluid analysis obtained during initial acute admission demonstrated normal protein, glucose and cell counts without evidence of infection. Alzheimer’s disease biomarkers (Aβ1-42, T-tau, P-tau) were not requested, as PCA had not been considered. Creutzfeldt-Jakob disease biomarkers (14-3-3 protein) were not obtained, given the prolonged course and absence of myoclonus. The patient later declined a repeat lumbar puncture when offered by our unit.

**Figure 1:**
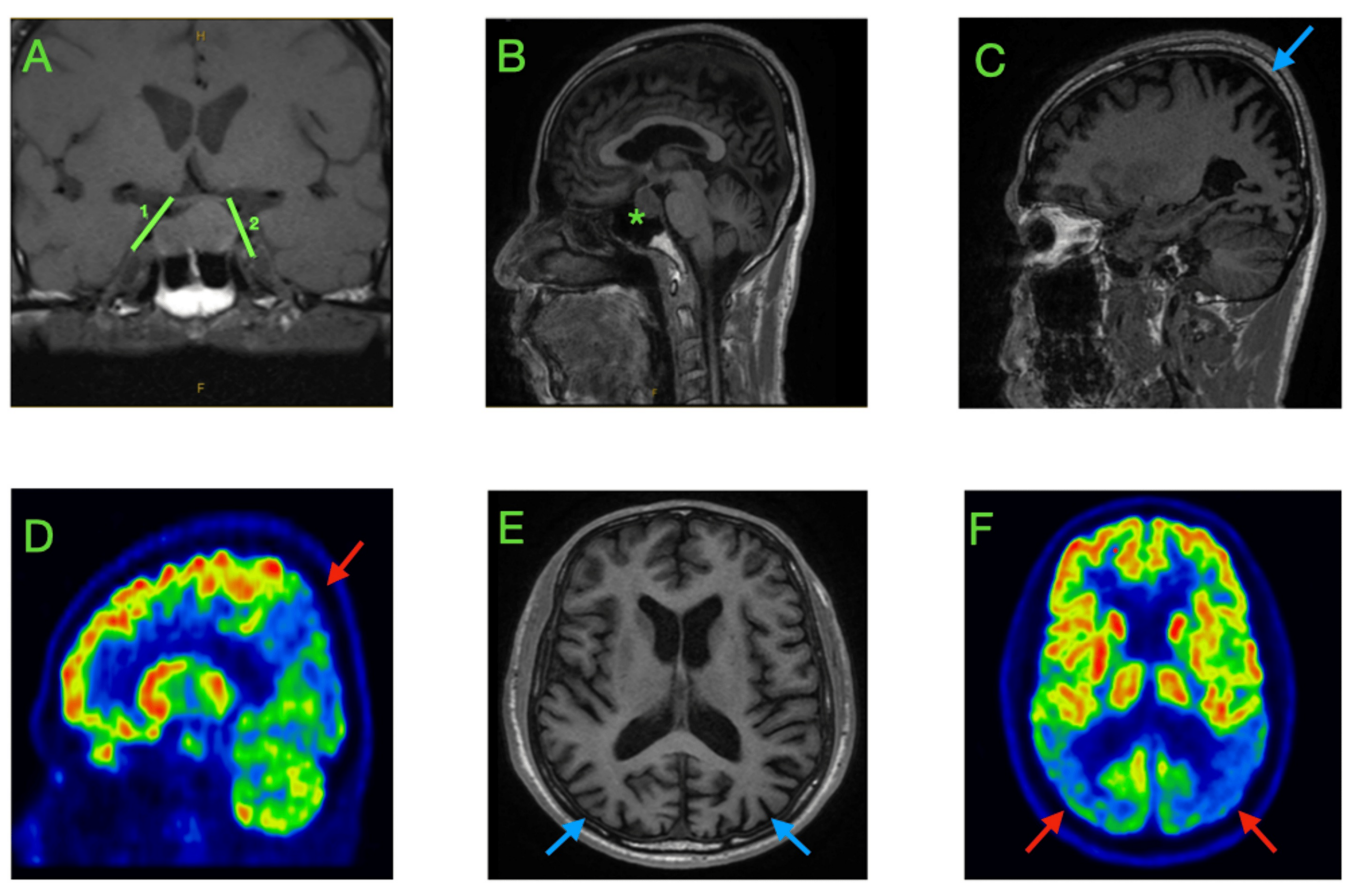
Magnetic resonance imaging and 18F-fluorodeoxyglucose positron emission tomography demonstrating pituitary macroadenoma and parieto-occipital changes. Coronal (A) and sagittal (B) T1-weighted magnetic resonance imaging (MRI) views demonstrate a pituitary macroadenoma (asterisk) measuring 14 x 17 mm (dimensions indicated in green). Sagittal MRI (C) demonstrates parietal cortical atrophy (blue arrow) with corresponding parieto-occipital hypometabolism on 18F-fluorodeoxyglucose positron emission tomography (FDG-PET) (D, red arrow). Transverse MRI (E) and FDG-PET (F) views similarly demonstrate parietal atrophy (blue arrows) and hypometabolism (red arrows). The posterior cortical changes support the diagnosis of posterior cortical atrophy, while the stable macroadenoma accounts for the right homonymous hemianopia.

MRI demonstrated a stable pituitary lesion measuring 14x17mm, bilateral hippocampal atrophy, and asymmetric parietal atrophy with preserved frontal volume (Figure 1A-1C, 1E). 18F-FDG-PET revealed prominent hypometabolism in the precuneus, posterior cingulate and occipital cortices (Figure 1D, 1F). Serial perimetry showed patchy, incongruent deficits with a preserved macular split, consistent with cortical visual dysfunction rather than fixed chiasmal compression.

Differential diagnosis

Alternative diagnoses were considered. PCA with dementia with Lewy bodies overlap was possible but considered less likely given the absence of Parkinsonism, cognitive fluctuations, rapid eye movement sleep disorder and frontotemporal hypometabolism on PET [2,10]. The visual hallucinations remained confined to his hemianopic fields, without altered frequency or evolving distress. We felt this was most consistent with Charles Bonnet syndrome, rather than a core feature of dementia with Lewy bodies [11]. Posterior reversible encephalopathy syndrome was excluded based on the absence of risk factors, typical clinical features, and radiological vasogenic oedema. Creutzfeldt-Jakob disease was considered unlikely given the prolonged course, absence of myoclonus, and lack of characteristic MRI signal abnormality [12], though 14-3-3 protein was not tested.

Management

Given the predominance of Alzheimer’s disease pathology underlying most PCA cases, an acetylcholinesterase inhibitor was commenced. Although evidence for pharmacotherapy in PCA is limited, treatment is typically extrapolated from Alzheimer’s disease studies, and case reports suggest potential cognitive and functional benefit [2,13]. Rivastigmine was initiated with planned titration. Additionally, an occupational therapy assessment was arranged to evaluate home safety and develop compensatory strategies for visuospatial deficits. Despite pharmacotherapy and multidisciplinary input, functional decline progressed, leading to a transition to residential aged care.

The pituitary lesion remains radiologically stable without neurosurgical intervention. He remains under geriatric, neurosurgical and endocrine follow-up.

## Discussion

Recognising cortical visual dysfunction in the presence of afferent disease

Distinguishing cortical from afferent visual impairment is especially challenging when both contribute to the clinical picture. Recent PCA case series note that diagnosis is often preceded by prolonged investigation for ocular or afferent disease, and highlight indicators that should prompt consideration of cortical pathology [5,14]. These include repeated presentation to optometry or ophthalmology, visual field deficits that are inconsistent with typical homonymous patterns, and functional complaints out of proportion to ocular examination findings. Posterior cortical deficits such as simultanagnosia may manifest as difficulty interpreting crowded letters on a Snellen chart or correctly discerning Ishihara plates. Correctly identifying this cortical deficit is difficult, and misdiagnosis as reduced visual acuity or colour blindness can occur [1,15]. Against this background, a pre-existing macroadenoma introduces further complexity: its anatomical plausibility naturally reinforces anchoring to an afferent explanation.

Our patient exemplifies these diagnostic pitfalls. The emergence of higher-order visual symptoms, disproportionate disability and inconsistent perimetry could have prompted reconsideration of a purely afferent explanation and raised suspicion of PCA earlier in the disease course.

A practical framework for evaluation of suspected dual pathology

**Figure 2:**
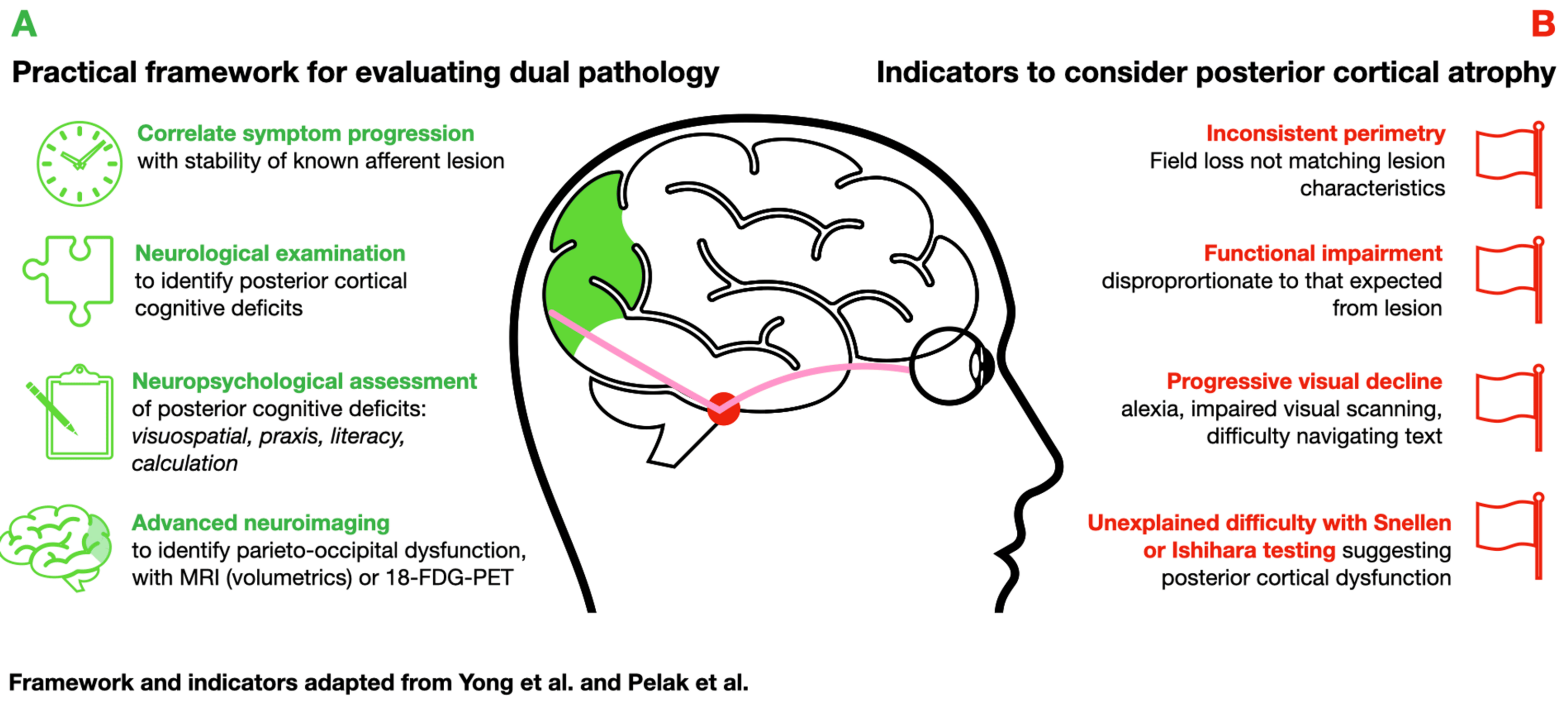
Framework for recognising posterior cortical atrophy in patients with established visual pathway pathology. (A) Key assessment domains for evaluating progressive visual symptoms when a structural lesion is known. (B) Clinical indicators that should prompt investigation for concurrent cortical disease. Centre panel: Schematic illustrating anatomical coexistence of posterior cortical atrophy (green) and a sellar mass compressing the optic chiasm (red); optic nerves, chiasm and tracts shown (pink). The illustration is stylised for conceptual clarity and was independently created by the authors. Framework adapted from Yong et al. [6] and Pelak et al. [15].

When patients with established afferent visual pathway disease develop worsening or progressive symptoms, a structured assessment is essential (summarised in Figure 2A). Evaluation begins by correlating symptom evolution with the stability of the known lesion on neuroimaging. A careful neurological examination should then assess for higher-order visual deficits that cannot be explained by field loss alone, for instance, Balint and Gerstmann syndromes, which are commonly identified during assessment [2, 14]. Neuropsychological evaluation is critical for characterising posterior cognitive dysfunction, with PCA-focused assessment tools for physicians and neuropsychologists currently undergoing refinement [15]. Advanced neuroimaging can identify characteristic parieto-occipital involvement, typically utilising MRI volumetric analysis or 18F-FDG-PET. Indicators of dual pathology, particularly progressive or inconsistent perimetry, should prompt investigation of concurrent cortical disease (summarised in Figure 2B). To support earlier recognition of PCA in eye-care settings, a rapid PCA-screening battery designed for optometrists and ophthalmologists is also in development [15].

Diagnostic criteria and biomarker considerations

PCA was diagnosed in this patient according to established consensus clinical criteria [2], which require an insidious onset and gradual progression, early visual impairment, at least three posterior cognitive deficits, and supportive neuroimaging evidencing predominant parieto-occipital atrophy or hypometabolism. This patient fulfilled each domain, demonstrating multiple posterior cognitive deficits, including constructional apraxia, space perception deficit, dressing apraxia, alexia, and features of both Gerstmann and Balint syndromes.

Post-mortem analyses of PCA have identified Alzheimer’s disease, corticobasal degeneration and Lewy body disease as the underlying the neuropathologies, though cases lacking typical features of recognised neurodegenerative disease also occur [3]. Alzheimer’s disease is the predominant cause, typically confirmed with histopathology or amyloid PET. In Alzheimer's disease, the correlation between amyloid biomarkers, treatment response and prognosis is well established [16,17], underpinning the emphasis on prospective biomarker assessment within the recently revised diagnostic framework [18]. Amyloid PET or CSF biomarkers are similarly recommended in the evaluation of suspected PCA to confirm underlying Alzheimer's pathology [2]. To date, studies of Alzheimer’s biomarkers in PCA have been underpowered to establish prognostic correlates [3]. In our case, CSF biomarkers were not obtained; the patient declined a repeat lumbar puncture after initial deferral. Whilst biomarker confirmation would have refined our differential diagnoses and strengthened diagnostic certainty, it would not have altered management or precluded referral to our regional multidisciplinary team. The specific underlying pathology, therefore, remains unconfirmed, though PCA itself is a clinical-radiological diagnosis, not contingent on biomarker confirmation.

## Conclusions

Delayed diagnosis is common in PCA. In this case, the pituitary lesion provided a plausible explanation for the patient’s decline and obscured recognition of the cortical degenerative process. Despite being pathophysiologically unrelated, the coexistence of a structural sellar lesion and PCA masked the underlying pathology.

Several learning points emerge from this case. In patients with established afferent disease, progressive or inconsistent visual findings, or deterioration discordant with stable imaging, dual pathology should be considered. The emergence of posterior cognitive deficits should heighten suspicion of PCA. Detailed neuropsychological assessment and multidisciplinary review are particularly valuable when cortical dysfunction is a possibility. More broadly, this case emphasises the importance of maintaining diagnostic flexibility. When symptom progression exceeds what a known lesion can explain, clinicians should remain alert to anchoring bias and consider alternative or concurrent pathology.
